# Depressive Symptoms After Mechanical Thrombectomy in Ischaemic Stroke: A Longitudinal Descriptive Study with Gender Differences

**DOI:** 10.3390/healthcare14172839

**Published:** 2026-09-03

**Authors:** Sara Ramírez Rozas, Maria Lara Martínez-Gimeno, Laura Carrasco San Andres, Francisco Gómez-Esquer, Jesús Esteban-Hernández, María-Gema Cid-Expósito

**Affiliations:** 1 Traumatology and Orthogeriatrics Unit, Ramón y Cajal University Hospital, Ctra. De Colmenar Viejo km 9.100, 28034 Madrid, Spain; 2 Program in Health Sciences, International Doctoral School, Rey Juan Carlos University, Av. de Atenas s/n, 28922 Alcorcón, Spain; 3 San Juan de Dios Foundation, 28016 Madrid, Spain; mlmartinez@comillas.edu; 4 Department of Health Sciences, San Juan de Dios School of Nursing and Physical Therapy, Comillas Pontifical University, 28350 Madrid, Spain; 5 SALBIS Research Group, Faculty of Health Sciences, University of León, 24401 Ponferrada, Spain; 6 Obstetrics Unit at Puerta de Hierro University Hospital, c/Manuel de Falla, nº1, 28222 Majadahonda, Spain; l.carrascosanandres@gmail.com; 7 Department of Basic Sciences, Faculty of Health Sciences, Rey Juan Carlos University, Avda. Atenas s/n, 28922 Alcorcón, Spain; francisco.gomez.esquer@urjc.es; 8 Consolidated Research Group on Anatomical, Molecular and Human Developmental Bases at Rey Juan Carlos University (GAMDES), 28922 Alcorcón, Spain; 9 Consolidated Research Group on Cognitive Neuroscience, Pain and Rehabilitation in Health Sciences, Rey Juan Carlos University, 28922 Alcorcón, Spain; 10 Department of Medical Specialties and Public Health, Faculty of Health Sciences, Rey Juan Carlos University, 28922 Alcorcón, Spain; jesus.esteban@urjc.es; 11 Department of Nursing and Stomatology, Faculty of Health Sciences, Rey Juan Carlos University, Avda. Atenas s/n, 28922 Alcorcón, Spain; gema.cid@urjc.es; 12 Research Group in the Pathophysiology and Pharmacology of the Digestive System, NeuGut, Rey Juan Carlos University, 28922 Alcorcón, Spain; 13 Nursing Care Research Group, Gregorio Marañón Institute of Health Research (IISGM), 28007 Madrid, Spain

**Keywords:** ischemic stroke, thrombectomy, depression, quality of life, sleep

## Abstract

**Introduction:** Stroke accounts for one-third of global mortality each year; in Spain, it is the second leading cause of death. One of the most commonly reported complications is post-stroke depression, which affects up to 30% of patients who suffer a stroke. When an ischaemic stroke occurs, the treatment of choice is mechanical thrombectomy; this is an endovascular procedure whereby the thrombus causing the ischemia is removed, thereby reducing sequelae, in most cases, which is why it is of interest to examine its influence on the development of post-stroke depression. This study aims to determine the proportions of depressive symptoms in patients with ischaemic stroke who underwent mechanical thrombectomy as a treatment option, and to explore potential gender differences in depressive symptoms over time. **Methods:** This was a multicentre, observational, descriptive, longitudinal, and prospective study, with follow-up at two weeks, one month, three months, and six months after the event. Depressive symptoms were analysed longitudinally and compared according to gender. **Results:** Patients who suffered an ischaemic stroke and underwent mechanical thrombectomy had a depressive symptom proportions of 11% at 15 days, decreasing to 7% at 6 months. No significant gender differences were observed in the proportions or trajectory of depressive symptoms during follow-up. A total of 92% of the sample showed no change in quality of life. **Conclusions:** Patients undergoing endovascular intervention for ischemic stroke showed a low prevalence of subsequent depressive symptoms, with no significant differences observed by gender.

## 1. Introduction

Stroke, defined as an interruption of cerebral blood flow, is a leading cause of death and long-term disability worldwide [1,2].

Although, globally, stroke is more common in men, it has been observed that women present with more severe cases, with a one-month mortality rate of 24.7% compared to 19.7% in men [3].

After coronary heart disease, stroke is the second leading cause of death worldwide [4] and the leading cause of disability; the most common sequelae are physical disability, speech and mobility disorders, and cognitive impairment [2].

Approximately 85% of strokes are ischaemic in origin, of which 40–45% are caused by a thrombotic event or occlusion of one of the major vessels [4,5].

When it comes to the loss of disability-adjusted life years, stroke is also the leading cause [6]; it has a significant impact on the quality of life of patients who suffer from it and on their families. A total of 30% of patients who survive a stroke will require long-term care, 20% will need assistance with walking, and 16% will require institutional care [1]. Stroke causes serious sequelae for patients in its acute phase, particularly in cases of severe ischaemic stroke requiring mechanical thrombectomy; many patients require admission to intensive care units due to neurological and hemodynamic instability, the need for close neurological monitoring, and, in some cases, ventilatory support. In this context, the patient is classified as a critical patient, which adds greater clinical and emotional complexity to the care process. It is regarded as a traumatic event, severely affecting the physical condition, but also entails psychological, social, and economic consequences, making it a serious global health problem [7,8].

Post-stroke depression is one of the complications that can arise following a stroke; it is reported that 30% of patients who suffer a stroke develop post-stroke depression [9,10]. It sets in after the onset of a stroke, sometimes in the short term (within 3 months) and, in other cases, later, appearing up to a year after the stroke [11]. One in three people who suffer a stroke will experience post-stroke depression, which has a significant impact on the long-term effects of this condition [12]. Recent evidence provides a deeper synthesis of its multifactorial etiology, highlighting several critical risk factors that drive its onset. Notably, functional impairment, particularly severe motor deficits and the loss of independence in activities of daily living, strongly predicts the development of depression. Additionally, sleep disturbances, including insomnia and sleep apnea, have been identified as major contributors that exacerbate neurological vulnerability and hinder cognitive and psychological recovery. These physical and neurological challenges are often compounded by psychosocial factors; specifically, a lack of robust social support from family or community networks significantly elevates the risk of depressive symptoms. Following the stroke itself, it is considered the most common psychiatric condition, making it vitally important to monitor patients’ psychological and functional well-being, as well as the impact on their quality of life [13,14].

Depression is a condition that leads to greater functional disability and a higher mortality rate, and is associated with a poorer quality of life [15], making functional recovery following a stroke more difficult [16].

Consequently, maximizing the success of acute interventions is a critical determinant not only for physical survival, but also for post-stroke mental health. In this context, advanced reperfusion therapies like mechanical thrombectomy play a pivotal role. The degree of functional recovery achieved by patients depends directly on the speed and effectiveness of the treatment provided. Interventions carried out as quickly as possible reduce subsequent complications and sequelae or even make them non-existent in some cases. From a clinical standpoint, the rapid neurological recovery facilitated by mechanical thrombectomy may be associated with a lower risk of depressive symptoms, mainly by preserving independent functioning and minimizing the profound existential shock of sudden disability. Full functional recovery is not always immediate, and this, combined with the social stigma associated with this condition, along with the resulting lifestyle changes, and despite the absence of serious sequelae, can trigger situations in which a depressive state develops on an emotional level [17].

It is important to note that it is not only functional status that influences the development of subsequent depression; severe physical sequelae are a component described among the predictors of subsequent depression, but they are not decisive. While successful revascularization may help lower depressive risk by improving functional outcomes, depression remains a complex, multifactorial phenomenon. Biological disruptions, pre-existing psychological vulnerabilities, and personal coping mechanisms mean that a positive neurological recovery does not entirely eliminate the risk of onset [18].

There are other risk factors that may influence the development of this condition, such as sleep quality [19] and its impact on quality of life, which make it important to assess the development of depressive symptoms without ruling out the various variables involved [20].

Furthermore, it is important to note that post-stroke depression significantly increases the risk of suffering a further stroke; patients have reduced mobility following the thrombotic event due to functional impairment, and the risk of thrombosis is higher [21].

In the acute phase of a stroke, particularly in patients admitted to intensive care units, it is difficult to make an accurate diagnosis of depressive symptoms; so, it is important to pay attention to the early symptoms. Patients tend not to recognize the symptoms caused by the mood disorder as characteristic of depression; they are commonly associated, by both patients and healthcare professionals, with the sequelae of the thrombotic event itself. Consequently, it is of great importance to screen stroke patients from the outset, using scales and on a serial basis, given the high incidence of depressive symptoms [22].

Mechanical thrombectomy is an established endovascular intervention for selected patients with acute ischaemic stroke due to large-vessel occlusion [23], an endovascular technique in which the thrombus is removed and blood flow is rapidly and effectively restored, facilitating the re-establishment of cerebral perfusion and significantly reducing subsequent sequelae. Moreover, considering the complex pathophysiological mechanisms underlying post-stroke depression, including neuroinflammatory processes, neurotransmitter imbalance, and neuronal damage, it is relevant to investigate whether rapid reperfusion therapies may influence the later development of depressive symptoms. However, there is limited literature specifically examining the descriptive association between the performance of mechanical thrombectomy and the onset of subsequent depression, as well as comparative evidence with other reperfusion treatments such as thrombolysis [24,25].

The main objective of this study is to determine the prevalence of depressive symptoms in patients with ischaemic stroke who have undergone mechanical thrombectomy as the treatment of choice and were treated at two high-complexity hospitals between December 2023 and December 2024.

Secondary objectives include describing the quality of life and functional capacity of patients who have suffered an ischaemic stroke and undergone mechanical thrombectomy as the treatment of choice, as well as exploring potential differences according to age, marital status, living arrangements, education, and physical activity level. We hypothesize that successful endovascular reperfusion is associated with a relatively low prevalence of subsequent depressive symptoms.

## 2. Materials and Methods

A multicentre, prospective, longitudinal, descriptive observational study was conducted with follow-up at 2 weeks, 1 month, 3 months, and 6 months after the stroke onset.

The target population consisted of patients who, following an ischaemic stroke, underwent mechanical thrombectomy at two specialist hospitals in Madrid. The sample was recruited over one year, from December 2023 to December 2024. With an estimated annual accessible population of 250 patients and assuming an expected proportion of post-stroke depression of 30%, (based on general post-stroke epidemiological literature, as specific post-thrombectomy estimates were lacking at the study design stage), the required sample size to estimate this proportion with a 95% confidence level and a precision of ±3 percentage points, applying the finite population correction, was calculated to be 196 patients. Assuming a potential 10% loss to follow-up, the target sample size was increased to 216 patients. However, data collection concluded at 200 patients at baseline. One participant passed away after the baseline assessment, and no additional loss to follow-up occurred during the subsequent telephone evaluations, resulting in a final sample of 199 participants at the three follow-up assessments. This sample size successfully retained the statistical precision initially planned (precision of ±3.1%).

The inclusion criteria considered were: patients who had suffered an ischaemic, non-hemorrhagic stroke; patients who were independent in activities of daily living before the stroke as documented in clinical records; no history of diagnosed depressive disorder or current antidepressant treatment; and patients who, following recanalization, had an adequate level of consciousness (Glasgow Coma Scale score of 15) to allow them to complete the scales.

The exclusion criteria were patients who suffered a hemorrhagic stroke during endovascular treatment; patients in whom complete revascularization of the occluded vessel was not achieved; and patients with extensive cerebral infarction resulting in lack of functional neurological recovery.

Consecutive non-probabilistic sampling was carried out in the two hospitals selected for this study, selecting patients as they were admitted to the neurointerventional unit for mechanical thrombectomy and after verifying that they met the inclusion criteria.

### 2.1. Variables

Depressive symptoms is the primary variable; the Patient Health Questionnaire-9 (PHQ-9) was used as the measurement tool. It consists of 9 questions, each scored from 0 to 3 points, with 0 being the minimum score (absence of depressive symptoms) and 27 the maximum (severe depressive symptoms). As a screening instrument for post-stroke depressive symptoms rather than a formal diagnostic assessment, a cut-off score of PHQ-9 ≥ 5 was used to define the presence of mild-to-severe depressive symptoms, in accordance with established screening thresholds. In addition, individual symptom items (such as PHQ-9 Item 2 for depressed mood/sadness) were explored as secondary indicators of core affective symptoms [26].

Functional capacity and quality of life were considered as secondary variables. Regarding functional capacity, the validated Barthel Index was used, comprising 10 items that measure basic activities of daily living, each scoring 0, 5, 10, or 15 points. The final score ranges from 0 to 100, with 0 indicating maximum dependence and 100 indicating independence [27].

Quality of life was measured using the ECVI-38 scale. It was validated in 2005 by Fernández-Concepción [28]. The scale comprises 38 items scored from 1 (no impact) to 5 (extreme impact), yielding a total raw score from 38 to 190, which is transformed into a 0–100 percentage scale (where higher scores indicate greater impairment). Four categories were defined based on the standardized score: severe impairment for scores of 75 or higher; moderate impairment between 50 and 74.9; mild impairment between 25 and 49.9; and no impairment for scores lower than 25 [29].

The health variables included in this study are physical activity, evaluated quantitatively as the self-reported hours of aerobic exercise performed per week prior to the stroke (as presented in Table 1), and sleep quality, as assessed using the Oviedo Sleep Questionnaire (OSQ), which has been validated against ICD-10 and DSM-IV diagnostic criteria in patients with depressive disorders; this questionnaire provides information on the sleep-wake cycle. It consists of 15 items, 2 of which relate to parasomnias and medication use; the other 13 are grouped into 3 subscales: subjective sleep satisfaction (item 1), insomnia (9 items), and hypersomnia (3 items). Each item is scored from 1 to 5, except for item 1, which is scored from 1 to 7. The insomnia subscale ranges from 9 to 45 points; higher scores indicate poorer sleep quality. The diagnosis of insomnia itself is established through items 2-1, 2-2, 2-3, 2-4, and 7: a categorical diagnosis is assigned when at least one of items 2-1 to 2-4 (difficulty falling asleep, difficulty maintaining sleep, early awakening, or non-restorative sleep) scores ≥ 3, indicating that the difficulty occurs on 3 or more days per week, together with item 7 (worry, tiredness, or reduced functioning due to the sleep difficulty) also scoring ≥ 3, i.e., occurring on 3 or more days per week. The remaining items provide additional relevant information on sleep [30].

The sociodemographic variables considered were age, gender, marital status, whether the participant had dependants, occupation, whether the job required specialized training, and level of education. Age gender and marital status were pre-specified as primary covariates to analyze potential differences in depressive symptom evolution.

After obtaining informed consent, participants were contacted by telephone to complete the study questionnaires. Responses were directly recorded and exported to a coded database.

### 2.2. Data Analysis

A descriptive analysis was carried out; the variables collected are qualitative, mostly nominal or ordinal, and the levels used were taken into account when interpreting the differences. To analyze the evolution of an ordinal categorical variable across time points within the same patients, a cumulative ordinal generalized linear mixed model with a logit link (CLMM) was applied.

The dependent variable was the ordinal variable resulting from categorizing the scale according to the established criteria, whilst the independent variable was the time of assessment. A random intercept per patient was included to model intra-individual correlation. Hospital center was evaluated as a random effect to control for potential cluster differences.

Where necessary, due to the low frequency of any level of the ordinal variable, adjacent categories were grouped to construct a binary variable, thereby simplifying the dependent variable and facilitating clinical interpretation. A generalized linear mixed model with a logit link (glmer) was fitted to this new dichotomous variable whilst also maintaining a random intercept per patient.

For the model examining the evolution of depressive symptoms specifically, the outcome was coded as a binary variable representing presence (mild, moderate, or severe symptoms combined) versus absence of depressive symptoms at each assessment time point, with Time Point A (2 weeks, baseline) used as the reference category. Model fit was assessed using standard diagnostics (AIC, BIC, log-likelihood, and deviance). The global effect of assessment time point was evaluated using a likelihood ratio test comparing the full model with a null model excluding the time point variable, using the anova() function in R version 4.4.1. All analyses were performed in the R statistical environment [31] using the ordinal [32] and lme4 [33] packages.

## 3. Results

The calculated sample size of 216 patients incorporated a 10% anticipated attrition rate to ensure a sufficient analytic sample. As all 200 recruited participants completed the questionnaires (100% completion rate), the effective analytic sample already exceeded the minimum target of approximately 194–195 participants required after accounting for expected loss-to-follow-up; recruitment was therefore concluded at n = 200 without the need to reach the original enrollment target of 216, which had accounted for an anticipated 10% attrition rate, distributed across centers as follows: 116 (58%) from Hospital Universitario Puerta de Hierro Majadahonda, comprising 57 women (59%) and 59 men (57%); and 84 (42%) from Hospital Universitario 12 de Octubre, comprising 40 women (41%) and 44 men (43%).

Regarding baseline demographic characteristics, substantial imbalances were observed between gender groups, which were subsequently identified as potential confounders for health outcomes. The mean age in the women’s group was 80 years (SD: 11.31), compared with 72 years in the men’s group (SD: 14.14) (*p* < 0.001).

Concerning marital status, there were statistically significant differences: 84% of the men’s group had a partner, compared with 46% of the women’s group (*p* < 0.001).

The percentage of people living alone is higher among women (33%) than among men (11%; *p* < 0.001).

About employment status, 156 people (78%) did not have a profession requiring higher education (both men and women); 64% of men are retired, compared to 73% of women who perform domestic work (0% in this employment category in the men’s group) (*p* < 0.001).

The group of women comprises 31% in the ‘no education’ category (compared to 8.7% of men), as well as lower percentages in both basic and higher education.

In terms of educational attainment, only 44 participants (22%) in the sample have a higher education qualification; of these, 30 are men (31%), and 13 are women (13%).

Concerning economic status, between 36–37% of men have incomes in the ranges of €1000–1499 and €1500–1999, compared with 33–35% of women who have net monthly incomes of less than €499 and less than €999, respectively. These baseline demographic, socioeconomic, and lifestyle factors were retained as covariates in subsequent adjusted gender outcome models.

Using the standardized cutoff of PHQ-9 ≥ 5 (indicating mild-to-severe depressive symptoms), the overall prevalence of depressive symptoms was 11% at two weeks, followed by a slight decrease to 8% at one month, 7.5% at three months, and 7% at six months (Table 2). Due to the small number of cases with depressive symptoms, the mild (5–9) and moderate (10–14) categories were grouped together and labelled as “mild/moderate” throughout the analysis. Although an omnibus test across all four follow-up periods indicated that this overall downward chronological trend did not reach statistical significance (*p* = 0.20), pairwise longitudinal evaluations revealed significant specific reductions between baseline and later follow-up points. Due to the low prevalence of depressive symptoms, quasi-complete separation was observed at Time Point A, resulting in an inflated intercept and correspondingly extreme odds ratios close to zero for this reference category (Table 3).

Taking gender into account, no statistically significant differences were observed in the prevalence of depressive symptoms across any of the time points (Table 4). Among men, 9.7% showed signs of depressive symptoms at two weeks, 5.8% at one month, and 4.9% at three and six months. In the group of women, the descriptive percentages were 11% at two weeks, 10% at one month and three months, and 9.3% at six months (Table 4). In multivariable logistic regression analysis adjusting for age, marital status, living arrangements, education, and physical activity level, gender was not an independent predictor of depressive symptoms at any follow-up interval.

Regarding the level of weekly aerobic exercise, 33% of men exercise for 3 h or more per week, compared with 10% of women; 86% of women and 55% of men do not engage in any weekly aerobic exercise.

Regarding sleep quality, unadjusted descriptive data showed that 10% of women reported sleep disturbances consistently across the four assessments, whereas prevalence among men remained low (0% at two weeks, 1.9% at one month, and 0% at three and six months; (*p* < 0.001). However, after adjusting for age, marital status, living arrangements, education, and physical activity level in multivariable models, the independent association between gender and sleep disturbances was markedly attenuated, demonstrating that baseline sociodemographic imbalances largely explained the observed raw differences between gender groups (Table 5).

Regarding functional and overall health status, cross-sectional evaluation showed high categorical stability across follow-up assessments. Specifically, 92% of participants remained within the non-impaired category for Quality of Life (ECVI-38) at all four data collection points. Similarly, evaluation via the Barthel Index indicated that 91% of participants maintained functional independence in basic activities of daily living across all four follow-up periods.

## 4. Discussion

Based on the data collected in this study, and taking into account the main variable, depressive symptoms in patients with ischaemic stroke who have undergone mechanical thrombectomy, the observed frequency of post-stroke depressive symptoms in this sample was lower than the approximately 30% prevalence reported in previous studies. Importantly, this lower frequency may equally be explained by our highly selected study population, which included patients with successful recanalization, preserved consciousness, no prior history of depression, and relatively favorable functional outcomes. Nevertheless, these findings should be interpreted with caution due to the absence of strong statistical significance and the potential influence of several sources of bias, including the non-probabilistic sampling method and the limited sample size, which may affect the generalizability of the results [8,10,14,34,35].

It is important to note that many of the participants answered question 2 of the PHQ-9 scale (have you felt down, depressed or hopeless?) with a score of 2 on numerous occasions, that is, they have felt this way on more than half the days, but to the remaining questions regarding sleep, appetite or fatigue, they answered ‘none of the days’, i.e., zero; so, the scale does not indicate the presence of a depressive state. However, this descriptive observation may reflect subclinical emotional, rather than a definitive depressive state, but only as an exploratory and non-conclusive analysis, and this interpretation remains speculative, as constructs such as fear of recurrence were not directly assessed in this study. While these findings carry implications for clinical nursing practice regarding close patient follow-up and the assessment of emotional well-being alongside standardized scales, they must be interpreted cautiously as they remain descriptive and some analyses did not reach statistical significance, since emotional distress following stroke may not always meet the diagnostic criteria for depression symptoms assessed by the PHQ-9 questionnaire [26,36].

When considering gender differences in depressive states, other studies have found that post-stroke depression occurs more frequently in women, and, although the factors have not yet been determined, these may be attributed to the social role women adopt in our society, biomedical causes (hormonal factors), and psychological causes [37,38]. In general, being a woman is identified as a risk factor for any type of depression, regardless of whether it follows a stroke or not, due to a complex multifactorial relationship. As described in some studies, women are up to 20% more likely to suffer from depression than men. It is important to note that a very high percentage of women are responsible for household care and lead a much more sedentary lifestyle, as this study’s results also indicate, although this remains a plausible but untested explanation in our cohort [39]. As for the other variables, when examining differences by gender, women have higher rates of poorer sleep quality, poorer functional status following a stroke, and, consequently, a lower quality of life, persisting for up to 6 months after the stroke, as observed in other studies. However, it must be emphasized that women in our cohort were substantially older than men (median 80 vs. 72 years) and exhibited marked baseline differences in partnership status, education, living arrangements, and physical activity levels; these baseline structural imbalances may account for or substantially confound the observed gender-related differences in sleep and quality of life. From a nursing perspective, the identification of women as a potentially more vulnerable group highlights the need for targeted follow-up strategies, including routine screening for emotional symptoms, sleep disturbances, and perceived quality of life. Such interventions may contribute to the early detection of patients at greater risk of psychological complications during recovery. The differences found in terms of gender are present in many of the measured variables suggesting a possible pattern of vulnerability among women, although not statistically confirmed, with higher percentages regarding depressive symptoms and sleep quality, amongst others, which are clear risk factors for the development of a depressive disorder. However, because no adjusted multivariate analyses were performed, these descriptive associations did not reach statistical significance, which is directly related to the limited sample size and variability among participants [3]. Therefore, these gender-based differences cannot be considered conclusive in our cohort [38,39].

It is important to note that the study participants showed a relatively limited impact on functional status during follow-up; however, due to the observational design and absence of a comparison group, these findings cannot be directly or solely attributed to mechanical thrombectomy; early restoration of cerebral circulation has been associated in previous studies with fewer complications and better quality-of-life outcomes, having a less pronounced impact on quality of life, as seen in the results. However, patients who are not treated in time or who have irreversible ischemia develop a greater number of sequelae that can undoubtedly influence their emotional and psychological state. Furthermore, the absence of a control group treated with other reperfusion therapies, such as thrombolysis alone, may also represent a limitation when interpreting the specific impact of mechanical thrombectomy on post-stroke depression [20,23,24].

Limitations and future directions: This study’s results have limitations related to sample size and characteristics, non-probability sampling, and telephone-based data collection, which may introduce potential selection and response biases, thereby affecting the generalizability of the findings. Another limitation of this study is the lack of data on clinically relevant variables such as NIHSS score, modified Rankin Scale, infarct location, recanalization grade, aphasia, rehabilitation status, and antidepressant treatment, as these were not collected within the scope of the original study protocol. Future studies incorporating these variables would allow for a more comprehensive interpretation of the relationship between depressive symptoms and clinical/functional outcomes after mechanical thrombectomy. The proportions of depressive symptoms following a stroke has been determined, and it might be worthwhile to conduct a longer-term follow-up to observe whether depressive symptoms develop over time, as there are studies that assess depressive states several years later [12]. Furthermore, excluding the most severely ill patients further reduces the possibility of generalization to the broader stroke population.

Crucially, the observational nature of this study design does not permit causal inference, and the complete absence of a concurrent comparison group (such as patients undergoing alternative reperfusion therapies or those with unsuccessful recanalization) precludes any definitive conclusions regarding the specific effect of mechanical thrombectomy on post-stroke depression. Furthermore, our findings are heavily constrained by a highly selected study population, which restricted inclusion to patients with successful reperfusion, preserved consciousness, no prior history of depression, and relatively favorable functional outcomes. This selective enrollment substantially limits the generalizability of our data to the broader, more severe stroke population and likely explains the low frequency of depressive symptoms observed in this cohort.

Additionally, methodological limitations regarding our analytical approach must be acknowledged. The use of strict PHQ-9 categorization thresholds may have led to a potential underestimation of subclinical depressive symptoms. The use of a self-administered screening instrument (PHQ-9) rather than a structured clinical diagnostic interview may have led to over- or under-identification of depressive symptoms, and this limitation should be considered when interpreting prevalence estimates. Furthermore, unadjusted demographic imbalances between gender, most notably the older age and distinct baseline sociodemographic profile of female participants, represent significant confounding factors that preclude drawing definitive subgroup conclusions without further multivariable control. Moreover, despite collecting a wide range of clinically relevant variables (such as sleep quality, demographics, and physical activity), the low proportions of depressive events in our sample prevented the execution of multivariate adjusted models to explore predisposing predictors without risking statistical underpowering and model over-fitting. Consequently, to avoid these statistical constraints, these variables had to be presented strictly as descriptive data. Additionally, the low overall prevalence of depressive symptoms led to quasi-complete separation in the mixed-effects logistic regression model used to assess the trend across time points (Table 3), reflected in a markedly inflated random-intercept variance and consequently extreme fixed-effect estimates at the reference time point. This should be considered when interpreting the magnitude of the corresponding odds ratios and confidence intervals.

To address these limitations and avoid the speculation inherent to purely observational data, future research should move beyond simply increasing the sample size alone. Instead, future efforts must focus on designing controlled, adequately powered longitudinal studies utilizing explicit control groups and encompassing broader, unselected, and more representative stroke populations to properly isolate therapeutic effects and evaluate multivariate predictors of post-stroke depression. In particular, future studies should incorporate structured clinical diagnostic interviews alongside standardized screening tools, apply adjusted multivariate models to disentangle age and sociodemographic confounding between sexes, and standardize PHQ-9 cut-off definitions to enable comparability across studies.

Furthermore, a psychometric limitation must be acknowledged regarding our instrument selection; certain assessment tools, such as the Oviedo Sleep Questionnaire, were not originally validated specifically within post-stroke populations, which may affect their contextual sensitivity and should be considered when interpreting the descriptive behavioral outcomes.

## 5. Conclusions

This study provides a valuable descriptive overview of psychological and functional outcomes six months following ischemic stroke in a specific cohort of patients. In this sample of patients treated with mechanical thrombectomy, the observed frequency of post-stroke depression symptoms (7%) was lower than that reported in the previous literature, while the vast majority of participants exhibited preserved functional independence in activities of daily living and an unaffected quality of life.

However, these favorable outcomes must be interpreted with strict caution and within the boundaries of our study design. Due to the purely observational nature of this research and the complete absence of a concurrent control group, no causal inferences or definitive conclusions can be drawn regarding the direct effect of mechanical thrombectomy on post-stroke depression. Furthermore, the low frequency of depressive symptoms observed here cannot be confidently attributed to the interventional procedure itself; instead, it is highly likely a reflection of our highly selected study population, which excluded individuals with prior depression, severe cognitive deficits, or poor initial recanalization.

Ultimately, while these descriptive parameters highlight a positive recovery trajectory within this specific subgroup, they underscore the critical need for future, adequately powered, controlled longitudinal studies. Such research is essential to truly isolate the therapeutic impacts of mechanical thrombectomy from selection biases and to comprehensively evaluate the multifactorial predictors of post-stroke mental health.

## Figures and Tables

**Table 1:** Baseline Demographic and Clinical Characteristics Stratified by Gender (N = 200).

Characteristic	Overall, N = 200	Female, N = 97	Male, N = 103	*p*-Value
Age, years	76 (64, 84)	80 (71, 87)	72 (61, 81)	0.001
Marital status				<0.001
With partner	132 (66%)	45 (46%)	87 (84%)	
Without partner	68 (34%)	52 (54%)	16 (16%)	
Living arrangements				<0.001
With independent partner	126 (63%)	46 (47%)	80 (78%)	
With dependent partner	5 (2.5%)	0 (0%)	5 (4.9%)	
With other independent persons	26 (13%)	19 (20%)	7 (6.8%)	
Alone	43 (22%)	32 (33%)	11 (11%)	
Education level				<0.001
Basic education	117 (59%)	54 (56%)	63 (61%)	
Higher education	44 (22%)	13 (13%)	31 (30%)	
No formal education	39 (20%)	30 (31%)	9 (8.7%)	
Physical activity level (aerobic exercise)				<0.001
1 h/week	7 (3.5%)	1 (1.0%)	6 (5.8%)	
2 h/week	9 (4.5%)	3 (3.1%)	6 (5.8%)	
3 or more h/week	44 (22%)	10 (10%)	34 (33%)	
None	140 (70%)	83 (86%)	57 (55%)	

**Table 2:** Evolution of Depressive Symptomatology Severity (PHQ-9) Across the Four Assessment Time Points (N = 200).

PHQ-9 Category	2 Weeks n (%)	1 Month n (%)	3 Months n (%)	6 Months n (%)
No depression (0–4)	179 (89.5)	183 (91.5)	184 (92.0)	186 (93.0)
Any depressive symptoms (5–27)	21 (10.5)	16 (8.0)	15 (7.5)	14 (7.0)
Missing data	0 (0.0)	1(0.5)	1 (0.5)	1 (0.5)
Total	200 (100.0)	199 (99.5)	199 (99.5)	199 (99.5)

Note: PHQ-9 = 9-item Patient Health Questionnaire. Percentages are calculated based on column totals.

**Table 3:** Logistic Regression Model Predicting Remission of Depressive Symptoms Across Time Points.

Term	Coeff.	Std. Error	Z	*p*	OR	95% Low CI	95% High CI
2 weeks (reference)	−10.701	1.333	−8.03	<0.001	0.00	0.00	0.00
1 month	−2.802	1.552	−1.81	0.071	0.06	0.00	1.27
3 months	−3.419	1.593	−2.15	0.032	0.03	0.00	0.74
6 months	−4.607	1.688	−2.73	0.006	0.01	0.00	0.27

Note: Coeff. = Estimated regression coefficient; Std. Error = Standard Error; OR = Odds Ratio; CI = Confidence Interval. Remission was defined as a PHQ-9 score < 5 at follow-up among participants with PHQ-9 ≥ 5 at baseline.

**Table 4:** Prevalence of Depressive Symptoms Stratified by Gender Across Assessment Time Points.

Assessment Time Point	PHQ-9 Category	Overall (N = 200) n (%)	Female (N = 97) n (%)	Male (N = 103) n (%)	*p*-Value *
2 weeks	No depression (0–4)	179 (89.5)	86 (88.7)	93 (90.3)	0.70
	Mild/moderate depressive symptoms (5–14)	21 (10.5)	11 (11.3)	10 (9.7)	
1 month	No depression (0–4)	183 (91.5)	86 (89.6)	97 (94.2)	0.20
	Mild/moderate depressive symptoms (5–14)	16 (8.0)	10 (10.4)	6 (5.8)	
	Missing data	0 (0.0)	0 (0.0)	0 (0.0)	
3 months	No depression (0–4)	184 (92.0)	86 (89.6)	98 (95.1)	0.14
	Mild/moderate depressive symptoms (5–14)	15 (7.5)	10 (10.4)	5 (4.9)	
	Missing data	0 (0.0)	0 (0.0)	0 (0.0)	
6 months	No depression (0–4)	186 (93.0)	88 (90.7)	98 (95.1)	0.20
	Mild/moderate depressive symptoms (5–14)	14 (7.0)	9 (9.3)	5 (4.9)	

Note: PHQ-9 = 9-item Patient Health Questionnaire. Percentages indicate the proportion within each gender sub-population at the specific time point. * *p*-value derived from Pearson’s Chi-square test or Fisher’s exact test comparing gender distributions across categorical responses within each time point.

**Table 5:** Longitudinal Distribution of Sleep Quality Categories across Follow-Up Time Points.

Sleep Category	2 Weeks n (%)	1 Month n (%)	3 Months n (%)	6 Months n (%)
Normal sleep	190 (95.0)	190 (95.0)	190 (95.0)	190 (95.0)
Sleep disturbance	10 (5.0)	9 (4.5)	9 (4.5)	9 (4.5)
Missing data	0 (0.0)	1 (0.5)	1 (0.5)	1 (0.5)
Total	200 (100.0)	199 (99.5)	199 (99.5)	199 (99.5)

Note: Percentages indicate column-wise proportions for each specific assessment phase. Sleep categories derived from standardized clinical screening scales.

## Data Availability

The data presented in this study are available as Supplementary Materials.

## Supplementary Materials

The following supporting information can be downloaded at: https://www.mdpi.com/article/10.3390/healthcare14172839/s1.

## Abbreviations

The following abbreviations are used in this manuscript:

PHQ-9	Patient Health Questionnaire-9
ECVI-38	Escala de Calidad de Vida en Ictus (versión de 38 ítems)
OSQ	Oviedo Sleep Questionnaire
ICD-10	International Classification of Diseases, 10th Revision
DSM-IV	Diagnostic and Statistical Manual of Mental Disorders, 4th Edition
CLMM	Cumulative Link Mixed Model
glmer	Generalized Linear Mixed Effects Regression (función del paquete lme4)
R	Lenguaje/entorno de programación estadística R

## References

[1] Soto Á., Guillén-Grima F., Morales G., Muñoz S., Aguinaga-Ontoso I., Fuentes-Aspe R. (2022). Prevalence and incidence of stroke in Europe: Systematic review and meta-analysis. An. Sist. Sanit. Navar..

[2] Rodríguez-Yañez M., Gómez-Choco M., López-Cancio E., Amaro S., Alonso de Leciñana M., Arenillas J.F., Ayo-Martín O., Castellanos M., Freijo M.M., García-Pastor A. (2021). Stroke prevention in patients with arterial hypertension: Recommendations of the Spanish Society of Neurology’s Stroke Study Group. Neurologia.

[3] Appelros P., Stegmayr B., Terent A. (2009). Sex differences in stroke epidemiology: A systematic review. Stroke.

[4] Amaya Pascasio L., Blanco Ruiz M., Milán Pinilla R., García Torrecillas J.M., Arjona Padillo A., Del Toro Pérez C., Martínez-Sánchez P. (2023). Stroke in Young Adults in Spain: Epidemiology and Risk Factors by Age. J. Pers. Med..

[5] Fuentes B., Amaro S., Alonso de Leciñana M., Arenillas J.F., Ayo-Martín O., Castellanos M., Freijo M., García-Pastor A., Gomis M., Choco M.G. (2021). Stroke prevention in patients with type 2 diabetes or prediabetes. Recommendations from the Cerebrovascular Diseases Study Group, Spanish Society of Neurology. Neurologia.

[6] Purroy F., Montalà N. (2021). Epidemiology of stroke in the last decade: A systematic review. Rev. Neurol..

[7] Hu R., Wang X., Liu Z., Hou J., Liu Y., Tu J., Jia M., Liu Y., Zhou H. (2022). Stigma, depression, and post-traumatic growth among Chinese stroke survivors: A longitudinal study examining patterns and correlations. Top. Stroke Rehabil..

[8] Taylor-Rowan M., Momoh O., Ayerbe L., Evans J.J., Stott D.J., Quinn T.J. (2019). Prevalence of pre-stroke depression and its association with post-stroke depression: A systematic review and meta-analysis. Psychological Medicine.

[9] Villa R.F., Ferrari F., Moretti A. (2018). Post-stroke depression: Mechanisms and pharmacological treatment. Pharmacology and Therapeutics.

[10] Das J., Rajanikant G.K. (2018). Post-stroke depression: The sequelae of cerebral stroke. Neuroscience and Biobehavioural Reviews.

[11] Ladwig S., Ziegler M., Südmeyer M., Werheid K. (2022). The Post-Stroke Depression Risk Scale (PoStDeRiS): Development of an Acute-Phase Prediction Model for Depression 6 Months After Stroke. J. Acad. Consult Liaison Psychiatry.

[12] Kang H.J., Bae K.Y., Kim S.W., Lee E.H., Kim J.T., Park M.S., Cho K.-H., Kim J.-M. (2018). Impact of acute-phase depression on functional outcomes in stroke patients over 1 year. Psychiatry Res..

[13] Kapoor A., Scott C., Lanctot K.L., Herrmann N., Murray B.J., Thorpe K.E., Lien K., Sicard M., Swartz R.H. (2019). Symptoms of depression and cognitive impairment in young adults after stroke/transient ischaemic attack. Psychiatry Res..

[14] Isuru A., Hapangama A., Ediriweera D., Samarasinghe L., Fonseka M., Ranawaka U. (2021). Prevalence and predictors of new-onset depression in the acute phase of stroke. Asian J. Psychiatr..

[15] Lee E.J., Kwon O.D., Kim S.J. (2022). Prevalence, awareness, and treatment of depression among community -dwelling stroke survivors in Korea. Sci. Rep..

[16] Davis J.C., Falck R.S., Best J.R., Chan P., Doherty S., Liu-Ambrose T. (2019). Examining the Inter-relations of Depression, Physical Function, and Cognition with Subjective Sleep Parameters among Stroke Survivors: A Cross-sectional Analysis. J. Stroke Cerebrovasc. Dis..

[17] Selvaraj S., Arora T., Casameni Montiel T., Grey I., Alfraih H., Fadipe M., Suchting R., Savitz S., Beauchamp J.E.S., Östlundh L. (2021). Early screening for post-stroke depression, and the effect on functional outcomes, quality of life, and mortality: A protocol for a systematic review and meta -analysis. BMJ Open.

[18] Zeng Y.Y., Cheng H.R., Cheng L., Huang G.Q., Bin C.Y., Tang W.J., He J. (2021). Comparison of post-stroke depression between acute ischaemic and hemorrhagic stroke patients. Int. J. Geriatr. Psychiatry.

[19] Silva L.C., Silva A., Rangel M.F.D.A., Caetano L.C.G., Teixeira-Salmela L.F., Scianni A.A. (2021). Depressive symptoms and functional status are associated with sleep quality after stroke. Top. Stroke Rehabil..

[20] Torrisi M., Bonanno L., Corallo F., Formica C., Giorgianni R., Marra A., Bramanti P., Arcadi F.A. (2022). The effect of intravenous thrombolytic therapy on post-stroke depression and cognitive dysfunction: A 3-month follow-up study. Appl. Neuropsychol. Adult.

[21] Dong L., Williams L.S., Brown D.L., Case E., Morgenstern L.B., Lisabeth L.D. (2021). Prevalence and course of depression during the first year after mild to moderate stroke. J. Am. Heart Assoc..

[22] Wu Q.E., Zhou A.M., Han Y.P., Liu Y.M., Yang Y., Wang X.M., Shi X. (2019). Post-stroke depression and risk of recurrent stroke: A meta-analysis of prospective studies. Medicine.

[23] Rengel M.D., Gil Romero J., De Freytas Rodriguez A., Sanchis Garcóa J.M., Guijarro Rosaleny J., Palmero da Cruz J. (2018). Mechanical thrombectomy in stroke: A retrospective analysis of one year’s experience. Intervencionismo.

[24] Roaldsen M.B., Jusufovic M., Berge E., Lindekleiv H. (2021). Endovascular thrombectomy and intra-arterial interventions for acute ischaemic stroke. Cochrane Database Syst. Rev..

[25] McCarthy D.J., Diaz A., Sheinberg D.L., Snelling B., Luther E.M., Chen S.H., Yavagal D.R., Peterson E.C., Starke R.M. (2019). Long -term outcomes of mechanical thrombectomy for stroke: A meta-analysis. Sci. World J..

[26] Morán M.O., Vega J.V., Aranguren I.G.C., Rodríguez J.A.R., Barraza E.W.L., Segovia J.L. (2024). Patient Health Questionnaire (PHQ-9): A Systematic Review and Meta-Analysis of Reliability and Generalizability.

[27] Ocagli H., Cella N., Stivanello L., Degan M., Canova C. (2021). The Barthel Index as an indicator of hospital outcomes: A retrospective cross-sectional study with healthcare data from older people. J. Adv. Nurs..

[28] Fernández-Concepción O., Ramírez-Pérez E., Álvarez M.A., Buergo-Zuáznabar M.A. (2008). Validation of the quality of life scale for stroke (ECVI-38). Rev. Neurol..

[29] Cardona-Arias J.A., Higuita-Gutiérrez L.F. (2014). Applications of a WHO-designed instrument for the quality of life evaluation. Rev. Cuba. Salud Publica.

[30] Barrera-Herrera A., Riffo-Álvarez C., Fernández-Hernández D., Ortiz-Peña F., Salazar-Fernández C. (2023). Oviedo Sleep Question-naire: Evidence of Validity and Reliability in Chilean University Students. Ibero—Am. J. Psychol. Diagn. Assess..

[31] R Core Team (2024). R: A Language and Environment for Statistical Computing.

[32] Bates D., Mächler M., Bolker B.M., Walker S.C. (2015). Fitting Linear Mixed-Effects Models Using lme4. J. Stat. Softw..

[33] Grabowska-Fudala B., Jaracz K., Górna K., Miechowicz I., Wojtasz I., Jaracz J., Kaźmierski R. (2018). Depressive symptoms in stroke patients treated and non-treated with intravenous thrombolytic therapy: A 1-year follow-up study. J. Neurol..

[34] Goyal M., Demchuk A.M., Menon B.K., Eesa M., Rempel J.L., Thornton J., Roy D., Jovin T.G., Willinsky R.A., Sapkota B.L. (2015). Randomized Assessment of Rapid Endovascular Treatment of Ischemic Stroke. N. Engl. J. Med..

[35] Carnes-Vendrell A., Deus J., Molina-Seguin J., Pifarré J., Purroy F. (2019). Depression and apathy after transient ischemic attack or minor stroke: Prevalence, evolution and predictors. Sci. Rep..

[36] Henares J., Ruiz-Pérez I., Sordo L. (2020). Mental health in Spain and differences by sex and by autonomous communities. Gac. Sanit..

[37] Dong L., Sánchez B.N., Skolarus L.E., Stulberg E., Morgenstern L.B., Lisabeth L.D. (2020). Sex difference in prevalence of depression after stroke. Neurology.

[38] Lee E.J., Kim J.S., Chang D.-I., Park J.H., Ahn S.H., Cha J.K., Heo J.H., Sohn S.-I., Lee B.-C., Kim D.-E. (2020). Depressive Symptoms in Stroke Patients: Are There Sex Differences?. Cerebrovasc. Dis..

[39] Schöttke H., Gerke L., Düsing R., Möllmann A. (2020). Post-stroke depression and functional impairments—A 3-year prospective study. Compr. Psychiatry.

